# Ascites-induced compression alters the peritoneal microenvironment and promotes metastatic success in ovarian cancer

**DOI:** 10.1038/s41598-020-68639-2

**Published:** 2020-07-17

**Authors:** Marwa Asem, Allison Young, Carlysa Oyama, Alejandro ClaureDeLaZerda, Yueying Liu, Matthew. J. Ravosa, Vijayalaxmi Gupta, Andrea Jewell, Dineo Khabele, M. Sharon Stack

**Affiliations:** 1 Department of Chemistry & Biochemistry, University of Notre Dame, Notre Dame, IN USA; 2 Harper Cancer Research Institute, University of Notre Dame, 1234 N. Notre Dame Ave., A200 Harper Hall, South Bend, IN 46617 USA; 3 Department of Biological Sciences, University of Notre Dame, Notre Dame, IN USA; 4 Department of Obstetrics & Gynecology, Medical Center, University of Kansas, Kansas City, USA

**Keywords:** Cancer, Cell biology, Cancer microenvironment, Gynaecological cancer

## Abstract

The majority of women with recurrent ovarian cancer (OvCa) develop malignant ascites with volumes that can reach > 2 L. The resulting elevation in intraperitoneal pressure (IPP), from normal values of 5 mmHg to as high as 22 mmHg, causes striking changes in the loading environment in the peritoneal cavity. The effect of ascites-induced changes in IPP on OvCa progression is largely unknown. Herein we model the functional consequences of ascites-induced compression on ovarian tumor cells and components of the peritoneal microenvironment using a panel of in vitro, ex vivo and in vivo assays. Results show that OvCa cell adhesion to the peritoneum was increased under compression. Moreover, compressive loads stimulated remodeling of peritoneal mesothelial cell surface ultrastructure via induction of tunneling nanotubes (TNT). TNT-mediated interaction between peritoneal mesothelial cells and OvCa cells was enhanced under compression and was accompanied by transport of mitochondria from mesothelial cells to OvCa cells. Additionally, peritoneal collagen fibers adopted a more linear anisotropic alignment under compression, a collagen signature commonly correlated with enhanced invasion in solid tumors. Collectively, these findings elucidate a new role for ascites-induced compression in promoting metastatic OvCa progression.

## Introduction

Among gynecologic malignancies, ovarian cancer (OvCa) has the highest mortality rate, resulting in ~ 14,000 deaths in 2019 in the United States alone. Early detection of OvCa is challenging, such that the majority (75%) of women present at diagnosis with metastatic disease. Direct extension and shedding into the peritoneal cavity are considered the main routes of metastatic dissemination. OvCa cells shed from the primary tumor are prevalent in the peritoneal cavity as single cells or multicellular aggregates, which subsequently colonize intra-peritoneal organs to form secondary lesions^1^. Malignant ascites is observed in many neoplasms with intra-peritoneal dissemination and is a common feature in OvCa. Many OvCa patients have ascites at the time of diagnosis, including some patients with early stage disease, and almost all patients with recurrent disease develop ascites^2–5^. Ascites accumulation results from increased vascular permeability accompanied by obstruction of the peritoneal lymphatics by disseminating cancer cells. Lymphatic drainage of accumulated fluids in the peritoneal cavity has been shown to slow from a normal rate of 200 mL/h to as low as 15 mL/h^2,5^. Moreover, ascites can reach volumes > 2 L, causing a striking elevation in intraperitoneal pressure (IPP) from normally sub-atmospheric 5 mmHg to as high as 22 mmHg, similar in magnitude to interstitial pressures measured in solid tumors^4,6–8^. Accumulated ascites can cause abdominal distension and discomfort, with the severity of symptoms correlated with the IPP^7^, and is considered a marker of poor prognosis. In addition to facilitating dissemination of OvCa cells in the peritoneal cavity, malignant ascites provides a nurturing environment for cancer growth, due to the presence of growth factors and bioactive lipids that enhance tumor cell proliferation^2,5,9^. Ascites is also rich in immune cellular components, which in turn augment the inflammatory environment through secretion of cytokines and chemokines, thereby accelerating disease progression^2,5^.

Ascites is targeted indirectly through pharmaceutical treatment and directly through fluid removal. In addition to reducing disease burden using platinum and taxol-based chemotherapy, diuretics and angiogenesis inhibitors such as bevacizumab are also employed in the management of ascites^10^. Ascites in patients with treatment-resistant disease is reduced through frequent paracentesis, however this temporary solution to relieve discomfort may lead to other complications such as visceral and vascular injury^2,5,11^.

The majority of OvCa patients (> 80%) develop peritoneal metastasis. The peritoneum, which consists of a monolayer of mesothelial cells (MC) on a vascularized connective tissue sub-mesothelial matrix that lines the abdominal cavity, covers the peritoneal organs with an overall surface area of 1.5-2m^2^. Metastasizing OvCa cells adhere to peritoneal MC, induce MC retraction and migrate, anchor and proliferate within the sub-mesothelial matrix to form secondary lesions. Previous studies have shown modification of peritoneal ultrastructure due to ascites accumulation^6,7^. Accumulated ascites also alters the loading environment in the peritoneal cavity, resulting in elevated compressive and shear stresses along peritoneal structures^12,13^. Interestingly, increased IPP, generated via CO_2_ pneumoperitoneum, is correlated with enhanced abdominal metastasis in a murine OvCa model^14^, suggesting that altered IPP can influence OvCa metastatic success.

While the contribution of the molecular and cellular components of ascites to OvCa progression is extensively studied^2^, data examining the direct impact of ascites-induced changes in IPP on OvCa progression are lacking. Experiments in this study were designed to test the hypothesis that increased compressive loading resulting from ascites accumulation modifies the peritoneal microenvironment, thereby enhancing metastatic success. Using a suite of in vitro, ex vivo and in vivo assays, we report that ascites induced-compression enhances OvCa adhesion to peritoneum, alters the peritoneal mesothelial surface ultrastructure and sub-mesothelial collagen alignment and induces tunneling nanotube (TNT) formation between OvCa cells and MC that enable mitochondria transport to OvCa cells. These results identify a novel role for ascites-induced compressive loading of cells and structures in the peritoneal microenvironment as a potentiator of metastatic progression.

## Results

### Artificial ascites-induced compression potentiates OvCa cell adhesion to peritoneum

**Figure 1:**
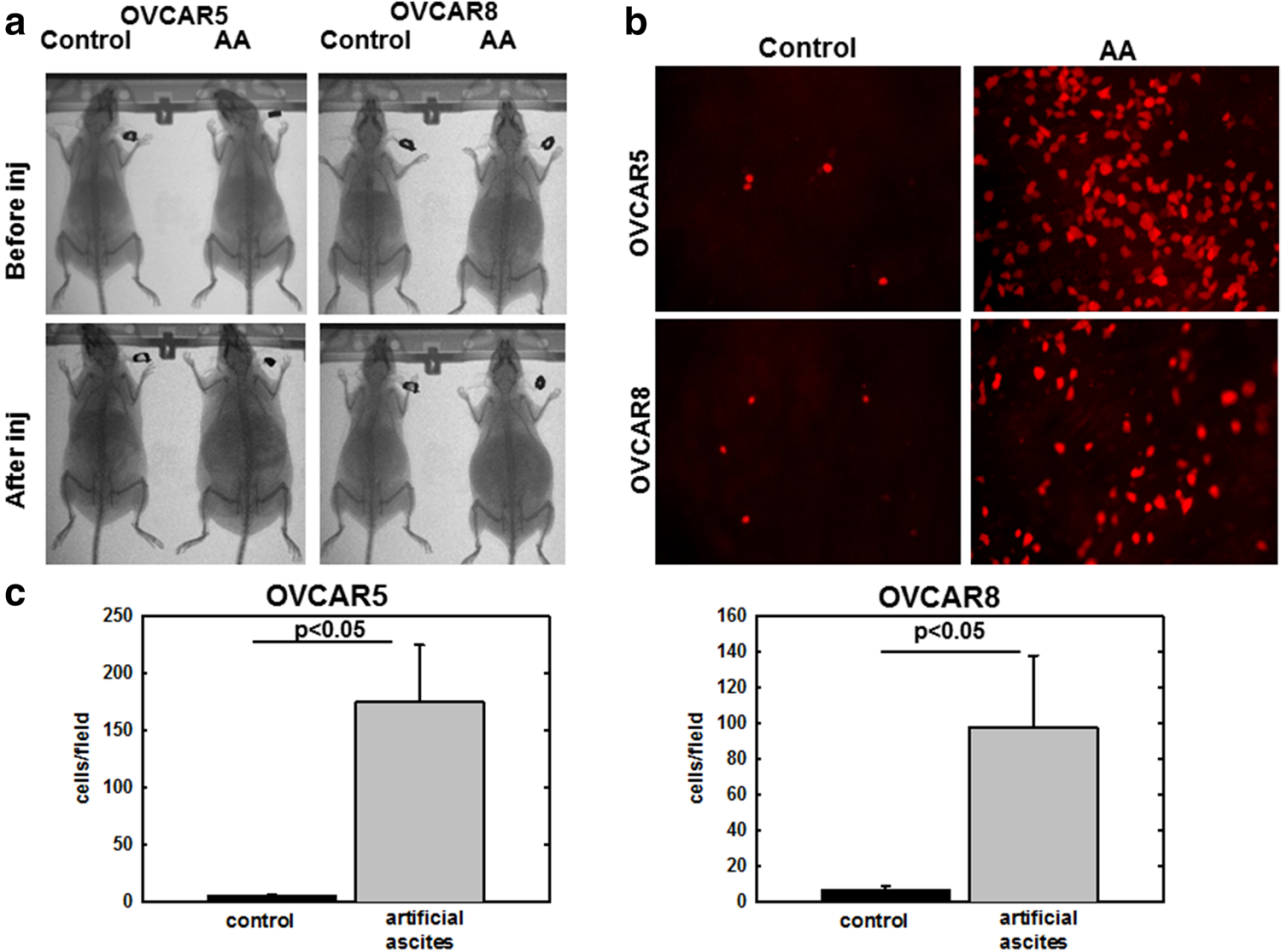
Artificial ascites model of compression enhances OvCa cell adhesion to peritoneum in vivo. (**A**) MicroCT Scans showing C57Bl/6 female mice injected i.p. with 1 mL (control) or 5 mL (artificial ascites, or AA) PBS containing 10^6^ RFP-tagged OVCAR5 or OVCAR8 cells, as indicated, for 5 or 8 h, respectively. (**B**) Mice were sacrificed, peritoneal tissue was collected and images of adherent cells to peritoneum were obtained using Echo Revolve fluorescent microscope at × 20 magnification. (**C**) Adherent cells were quantified using ImageJ. All experiments in were performed as triplicates with three independent biological replicates per cell line. All results are presented as mean ± s.e.m. and P-values were calculated using a Student’s two-tailed t-test. P < 0.05 is statistically significant.

The peritoneal MC monolayer represents a barrier to OvCa cell access to the sub-mesothelial extracellular matrix, wherein OvCa cells anchor and proliferate to form secondary lesions^15^. Successful adhesion of OvCa cells to the peritoneal surface induces mesothelial cell retraction and exposure of the sub-mesothelial matrix^16–19^. A previous study showed that elevated IPP (via CO_2_ pneumoperitoneum) in a murine OvCa model enhanced peritoneal metastases^14^. To investigate whether ascites-induced IPP influences early events in adhesion of OvCa cells to peritoneum, an in vivo artificial ascites assay was developed in which C57Bl/6 female mice were injected i.p. with RFP-tagged OVCAR5 or OVCAR8 cells in a large volume (5 mL) of PBS to mimic the ascites condition, relative to control mice that received tumor cells in a small volume (1 mL) of PBS (Fig. 1a)^20^. After allowing time for tumor cell i.p. adhesion (5–8 h), mice were euthanized and the peritoneum was dissected and imaged to enable quantitation of adherent tumor cells (Fig. 1b). Adhesion to omental tissue was rapid (30 min–1 h) and did not differ between control and artificial ascites conditions (data not shown). However a 20–30 fold increase in adhesion of both OVCAR5 and OVCAR8 cells to peritoneal surfaces in vivo was observed in mice with artificial ascites compared to control mice (Fig. 1c), suggesting that fluid induced changes in IPP enhance peritoneal metastasis through potentiating OvCa cell adhesion.

### Compression alters peritoneal mesothelial cell surface ultrastructure and induces TNT formation

**Figure 2:**
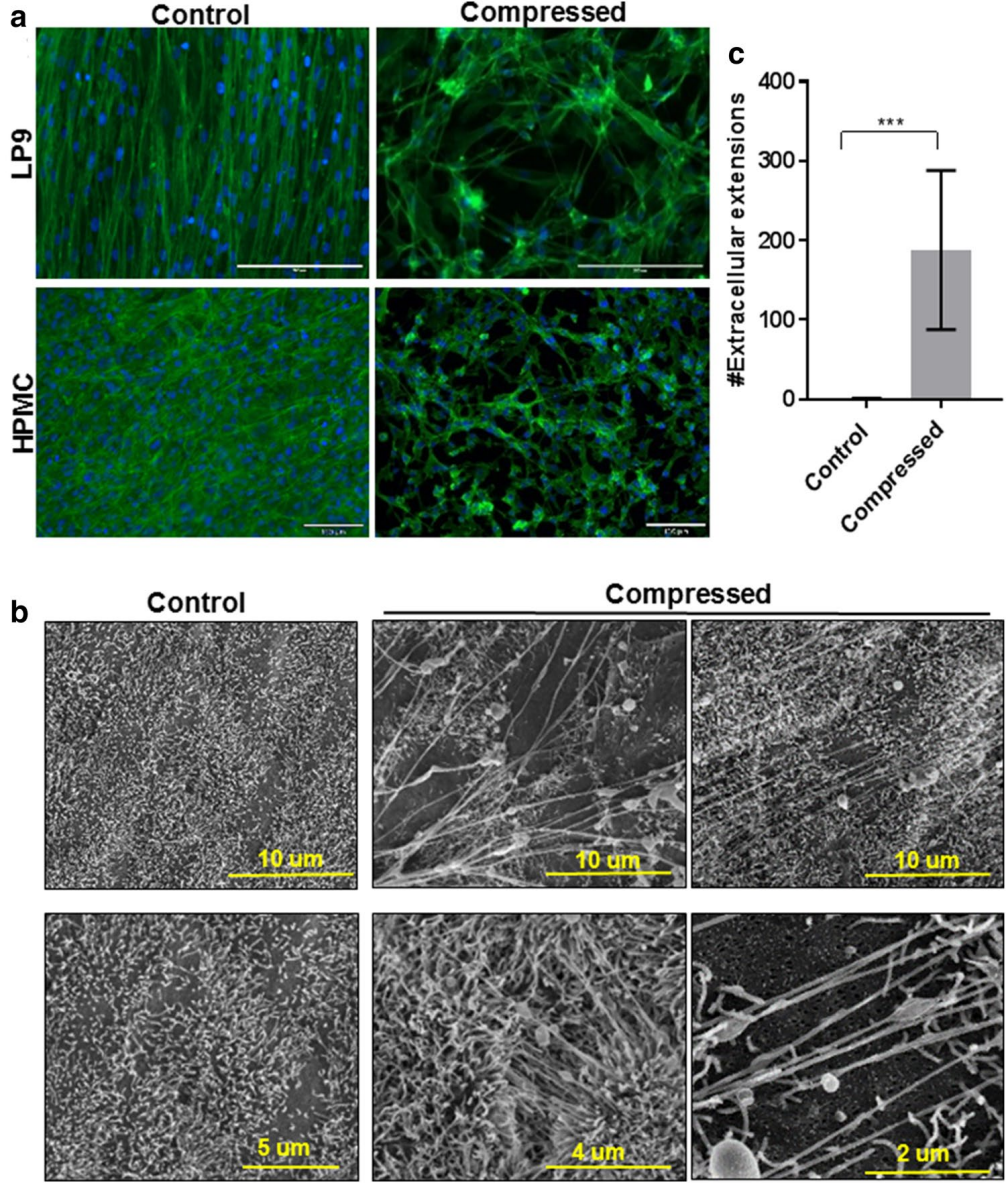
Compression alters peritoneal mesothelial morphology and surface ultrastructure in vitro and ex vivo. **(A)** LP9 human peritoneal mesothelial cells or primary human mesothelial cells (HPMC) were cultured in control or compressed conditions using a Flexcell Compression Plus System (~ 3 kPa; ~ 22 mmHg) for 24 h. Cells were fixed with 4% PFA buffer and stained with Phalloidin488 and DAPI. Cells were imaged with Leica DM5500 fluorescence microscope at × 20 magnification. Scale bar, 200, 130 µm. (**B**) Murine peritoneal explants were maintained ex vivo under control or compressed conditions (~ 3 kPa; ~ 22 mmHg) for 1 h. Explants were fixed and processed for imaging by FEI-Magellan 400 Field Emission Scanning Electron Microscope. Scale bar, 10, 5, 4, 2 µm (**C**) Tunneling nanotubes (TNT) were quantified using ImageJ. Three mice were included in each group with 10 images analyzed for each mouse. All results are presented as mean ± s.e.m. and *P*-values were calculated using a Student’s two-tailed *t*-test. Triple asterisk indicates significant p-value < 0.01.

During OvCa progression, peritoneal MC undergo morphological and molecular modifications that contribute to disease progression^21^. As the MC monolayer lines the inner surface of the peritoneum and covers peritoneal organs, these cells are directly subjected to ascites-induced compression. To evaluate whether ascites-induced compression alters MC morphology or ultrastructure, we used a Flexcell Compression Plus System to apply controlled compressive force (~ 3 kPa; ~ 22 mmHg, comparable to IPP in OvCa patients with tense ascites) to a human peritoneal MC cell line (LP9) or to primary human peritoneal MC (HPMC)^7,8^. Strikingly, compressed LP9 and HPMC cells exhibited dramatic changes in morphology, as evidenced by retraction, acquisition of a more mesenchymal phenotype and formation of sub-micrometer scale intercellular projections (Fig. 2a). Similar results were seen when intact murine peritoneal tissue explants were subjected to compression (~ 3 kPa; ~ 22 mmHg) ex vivo. Scanning electron microscopy (SEM) analysis of the compressed peritoneum showed alterations in the mesothelial surface consistent with the formation of tunneling nanotubes (TNT) connecting distant MC (Fig. 2b). The compression-induced TNTs are actin-based, have a length of ~ 3-100 µm (Fig. 2c), a diameter of 30-60 nm and are not attached to the surrounding substratum^22,23^.

**Figure 3:**
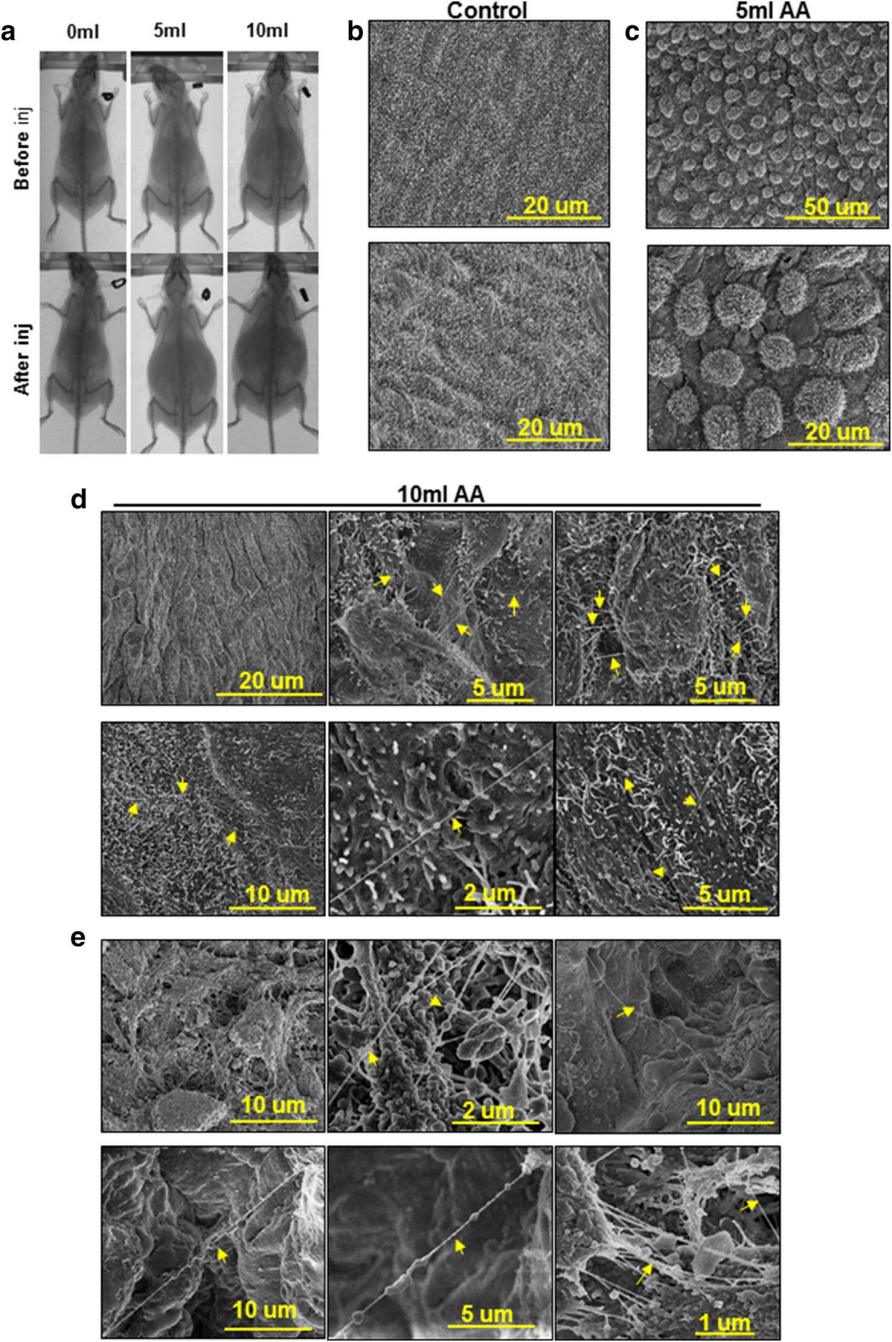
Artificial ascites model of compression alters peritoneal mesothelial cell morphology and surface ultrastructure in vivo. (**A**) C57Bl/6 female mice were injected i.p. with 5 or 10 mL PBS to model artificial ascites. MicroCT images of mice were taken before and after PBS injection. Peritoneal explants were collected from (**B**) control mice, (**C**) mice with 5 mL of artificial ascites, or (**D**) mice with 10 ml of artificial ascites. Explants were fixed and processed for imaging using a FEI-Magellan 400 Field Emission Scanning Electron Microscope. Yellow arrows indicate TNT. (**E**) Human peritoneal samples from women with ovarian cancer with ascites (n = 3) were fixed and processed for imaging of the mesothelial surface using a FEI-Magellan 400 Field Emission Scanning Electron Microscope. Yellow arrows indicate TNT.

To further evaluate compression-induced TNT formation in vivo, C57Bl/6 female mice were injected i.p with either 5 or 10 mL of PBS for 5 h (Fig. 3a)^20^, euthanized and the peritoneal tissue was dissected and processed for SEM imaging. Retraction of MC was observed in mice injected with 5 mL PBS relative to control mice (Fig. 3b, c). When injected with a higher fluid volume to mimic tense ascites (10 mL), abundant TNT formation was observed on the peritoneal mesothelial surface (Fig. 3d), suggesting a pressure threshold required for induction of TNT formation in vivo. Similar to structures induced by injection of high volume artificial ascites in mice, peritoneal tissue obtained from human OvCa patients with ascites displayed remarkably similar TNT structures (Fig. 3e).

**Figure 4:**
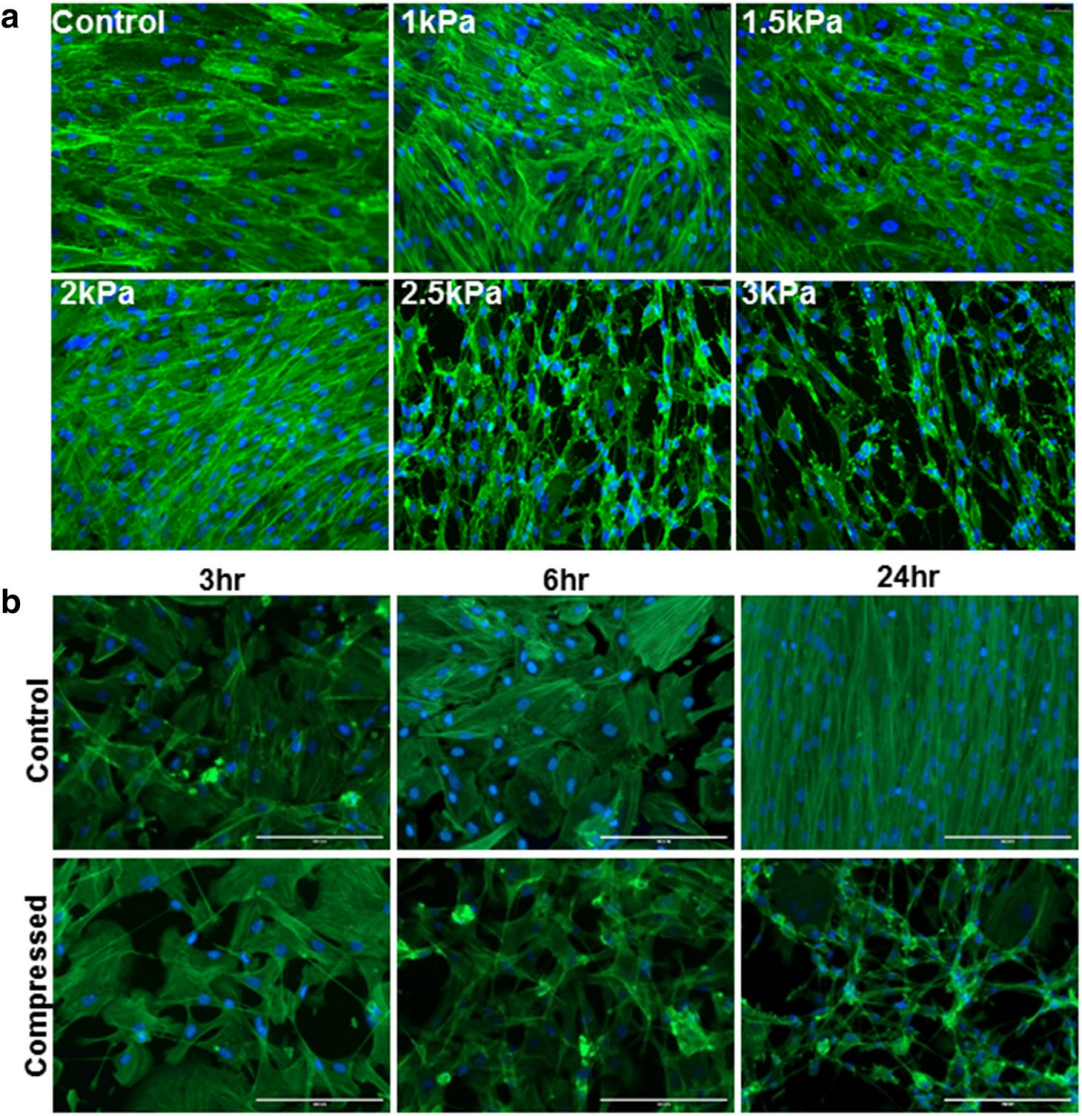
Evaluation of threshold parameters for compression-induced formation of nanoscale cell surface projections. (**A**) LP9 human peritoneal mesothelial cells were cultured atop type I collagen and subjected to a gradient of compressive force (1–3 kPa as indicated; 7.5–22.5 mmHg) for 24 h. Cells were fixed with 4% PFA buffer and stained with Phalloidin488 and DAPI. Cells were imaged with Leica DM5500 fluorescence microscope at × 20 magnification. Scale bar, 130 µm (**B**) LP9 cells cultured as described above were compressed at 3 kPa (~ 22 mmHg) for the time points indicated. Cells were fixed with 4% PFA buffer, stained with Phalloidin488 and DAPI and imaged with Leica DM5500 fluorescence microscope at 20 × magnification. Scale bar, 200 µm.

To assess the minimum threshold required to form TNT in peritoneal MC, a compressive gradient (1–3 kPa; 7.5–22 mmHg) was applied to LP9 cells using the Flexcell Compression Plus System. Under these conditions, a minimum pressure of 2.5 kPa (~ 18.5 mmHg) was needed to induce TNT formation in LP9 cells (Fig. 4a). Furthermore, TNT formation was observed as early as 3 h in compressed LP9 cells (~ 3 kPa; ~ 22 mmHg) (Fig. 4b). Together, these data highlight a novel effect of ascites-induced compression on peritoneal ultrastructure, via alteration of mesothelial surface morphology and induction of TNT formation.

### Compression promotes TNT formation between OvCa cells and peritoneal MCs

**Figure 5:**
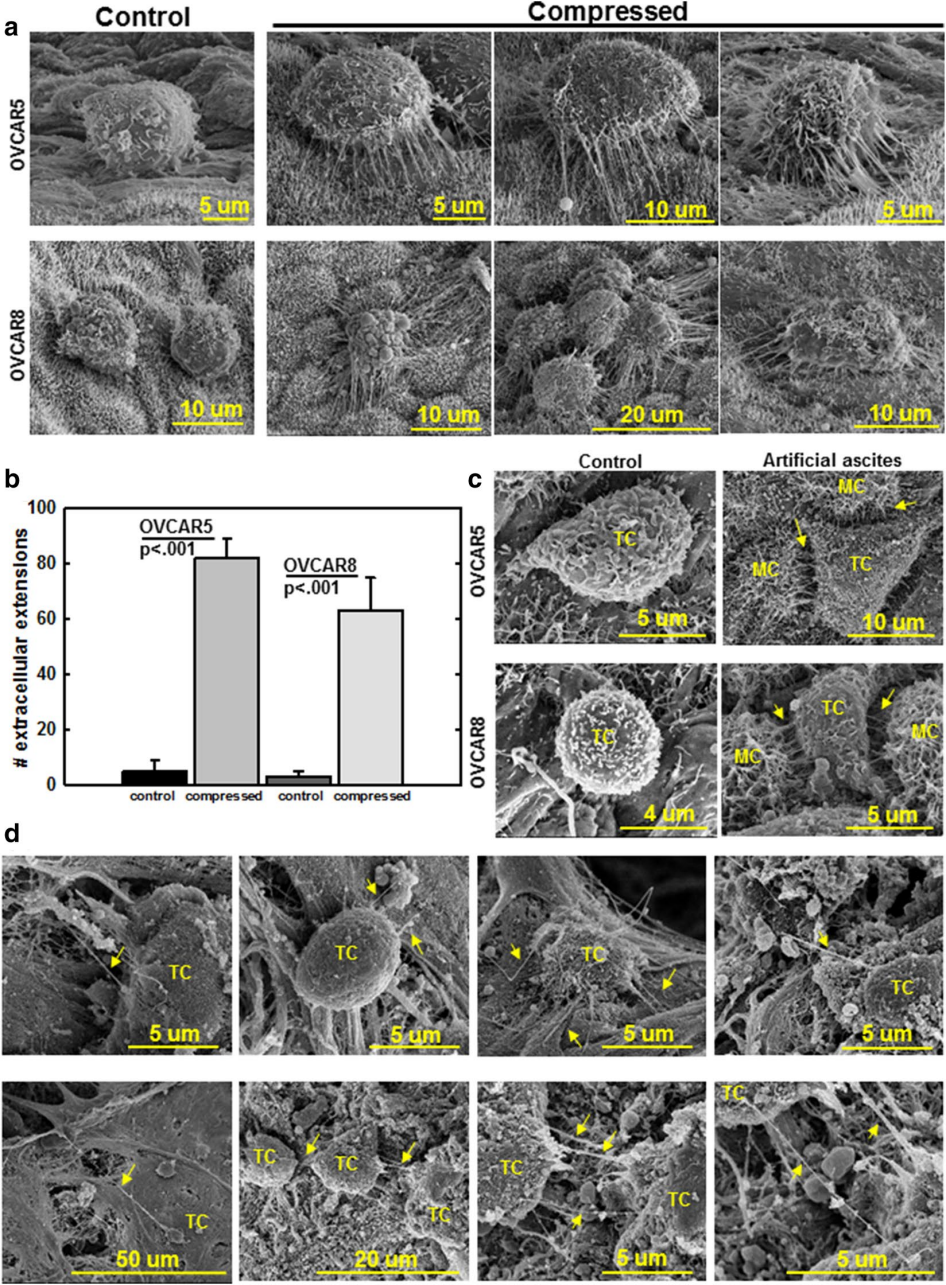
Compression enhances the formation of nanoscale projections between OvCa cells and peritoneal MCs. (**A**) OVCAR5 or OVCAR8 cells were added atop the mesothelial surface of murine peritoneal explants ex vivo followed by compression (~ 3 kPa; ~ 22 mmHg) for 30 min. The peritoneal explants were fixed and processed for imaging by FEI-Magellan 400 Field Emission Scanning Electron Microscope. (**B**) Quantification of nanotubes formed between OVCAR5 or OVCAR8 cells and murine peritoneal mesothelial surface. (**C**) C57Bl/6 female mice were injected i.p. with 1 mL (control) or 10 ml (artificial ascites) PBS containing 10^6^ OVCAR5 or OVCAR8 cells in an in vivo artificial ascites experiment. Peritoneal explants were collected, fixed and processed for imaging by FEI-Magellan 400 Field Emission Scanning Electron Microscope. *TC* tumor cell, MC: mesothelial cell. (**D**) Human peritoneum samples containing OvCa metastases were fixed and processed for imaging by FEI-Magellan 400 Field Emission Scanning Electron Microscope. *TC* tumor cell, *MC* mesothelial cell. Yellow arrows refer to TNT.

As adhesion of OvCa cells to the mesothelial surface is a key event in metastasis, we sought to model how ascites-induced compression may impact OvCa cell adhesion to peritoneal mesothelium. Compression (~ 3 kPa; ~ 22 mmHg) was applied to OVCAR5 or OVCAR8 cells in contact with murine peritoneal explants ex vivo, using the Flexcell Compression Plus System. Strikingly, both compressed OVCAR5 and OVCAR8 cells formed extensive nanoscale projections contacting the peritoneal mesothelial surface as early as 30 min (Fig. 5a, b). As previous data showed high level expression of the non-canonical Wnt ligand Wnt5a in OvCa ascites^9^, the effect of compression on Wnt5a expression was examined. Compression upregulated expression of *WNT5A* mRNA and Wnt5a protein (Suppl. Fig. 1a, b). Addition of exogenous Wnt5a to OvCa cells induced formation of nanoscale projections visible by fluorescence and scanning electron microscopy (Suppl. Fig. 1c, d), suggesting a role of Wnt5a signaling in TNT formation. In an in vivo model, TNT formation with peritoneal mesothelial cells was observed in an artificial ascites mouse model injected with either OVCAR5 or OVCAR8 cells (Fig. 5c). Similar to the murine in vivo model, human peritoneal tissues with metastatic lesions, collected from OvCa patients and imaged with SEM, also exhibited TNT between tumor cells and the peritoneal mesothelial surface (Fig. 5d).

**Figure 6:**
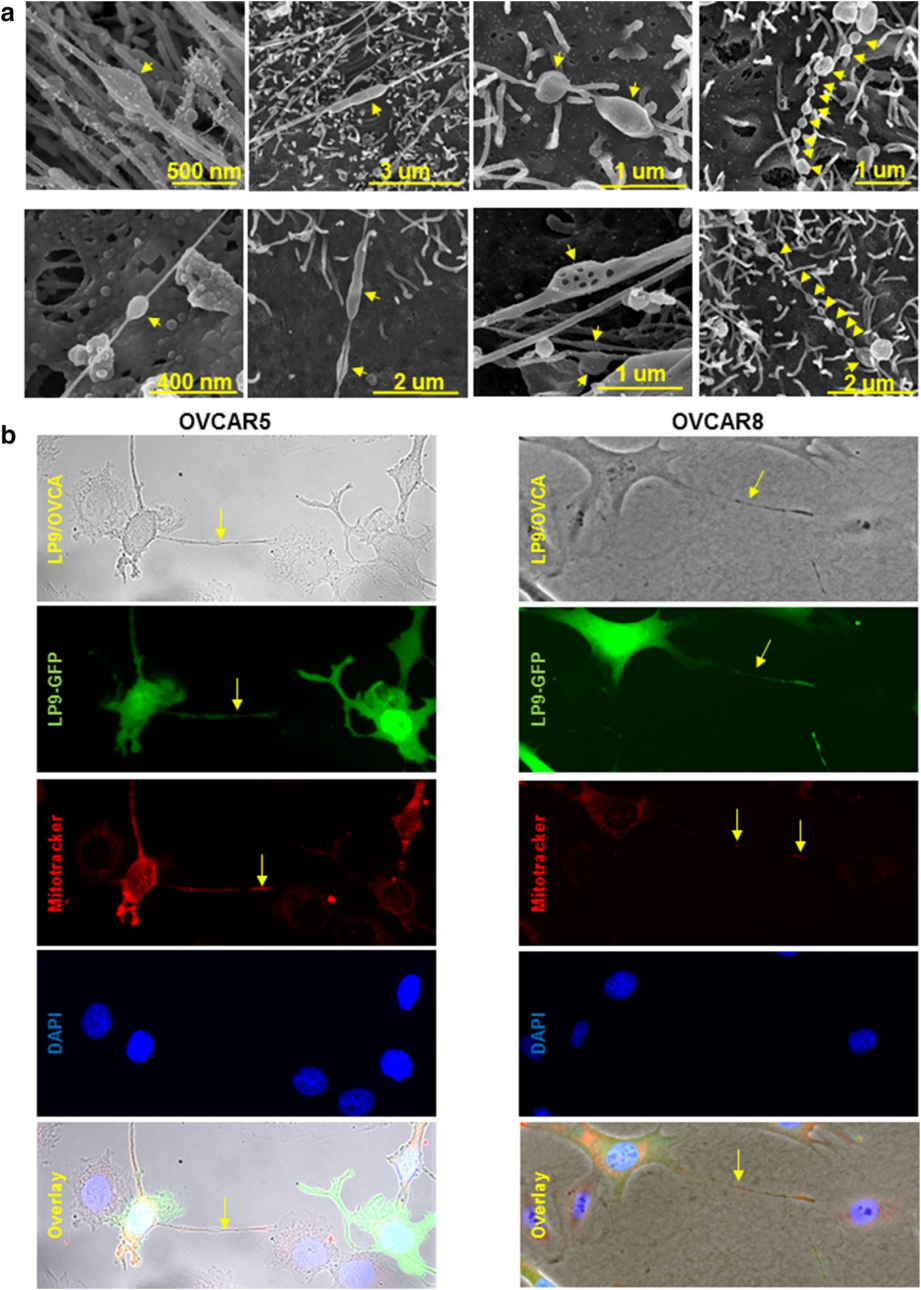
Compression-induced nanotubes adopt distinct morphologies and participate in mitochondria transport between LP9 mesothelial cells and OvCa cells. (**A**) High magnification scanning electron micrographs of TNT formed under compression (~ 3 kPa; ~ 22 mmHg) in murine peritoneal explants. The peritoneal explants were compressed ex vivo for 1 h, fixed and processed for imaging using a FEI-Magellan 400 Field Emission Scanning Electron Microscope. Yellow arrows refer to distensions in the nanotubes. (**B**) GFP-tagged LP9 human peritoneal mesothelial cells were incubated with MitoTracker red to label mitochondria, then co-cultured with OVCAR5 or OVCAR8 cells under compression (~ 3 kPa; ~ 22 mmHg) for 24 h and stained with DAPI. Cells were imaged with Leica DM5500 fluorescence microscope at × 20 magnification. Top panel: phase image showing LP9 and OVCAR cells; second panel: GFP-tagged LP9 peritoneal MC; third panel: MitoTracker red showing labeled mitochondria in LP9 cells and extracellular projections; fourth panel: DAPI-stained nuclei of both LP9 and OVCAR cells; fifth panel: overlay. Yellow arrows denote nanotubes or labeled mitochondria transferred in nanotubes.

It has been previously demonstrated that TNTs mediate the transfer of cellular cargo and organelles between cells under mechanical stress^22,23^. Interestingly, the compression-induced TNTs exhibit variations in the number and shape of distensions in the nanotube, suggesting a transfer of cargo between compressed cells (Fig. 6a). As mitochondrial transport through TNT has been reported in several malignant cells^24–28^, we investigated the possibility of mitochondrial transport in the compression-induced TNTs using GFP-tagged LP9 human MC with fluorescently labeled mitochondria (MitoTracker Red). These LP9 cells were then cultured with either OVCAR5 or OVCAR8 cells under compression (~ 3 kPa; ~ 22 mmHg). TNTs containing fluorescent (red) mitochondria were observed in nanotubes formed between MC and both OVCAR5 and OVCAR8 cells (Fig. 6b), indicating the transport of mitochondria from LP9 cells to OvCa cells.

### Compression alters peritoneal collagen fiber alignment

Disseminating OvCa cells adhere to the peritoneal mesothelial surface, intercalate within the MC layer, and induce MC retraction to facilitate invasion of the underlying type I collagen-rich ECM, wherein they proliferate to form secondary lesions^11,16,17,19^. In the ECM of normal tissues, collagen fibers are wavy and isotropic^29–31^. During cancer progression, collagen fibers are realigned, becoming straightened and anisotropic. This alteration in collagen quaternary structure has been shown to enhance cancer cell motility and invasion, leading to tumor progression and poor survival in several neoplasms^29,32^.

**Figure 7:**
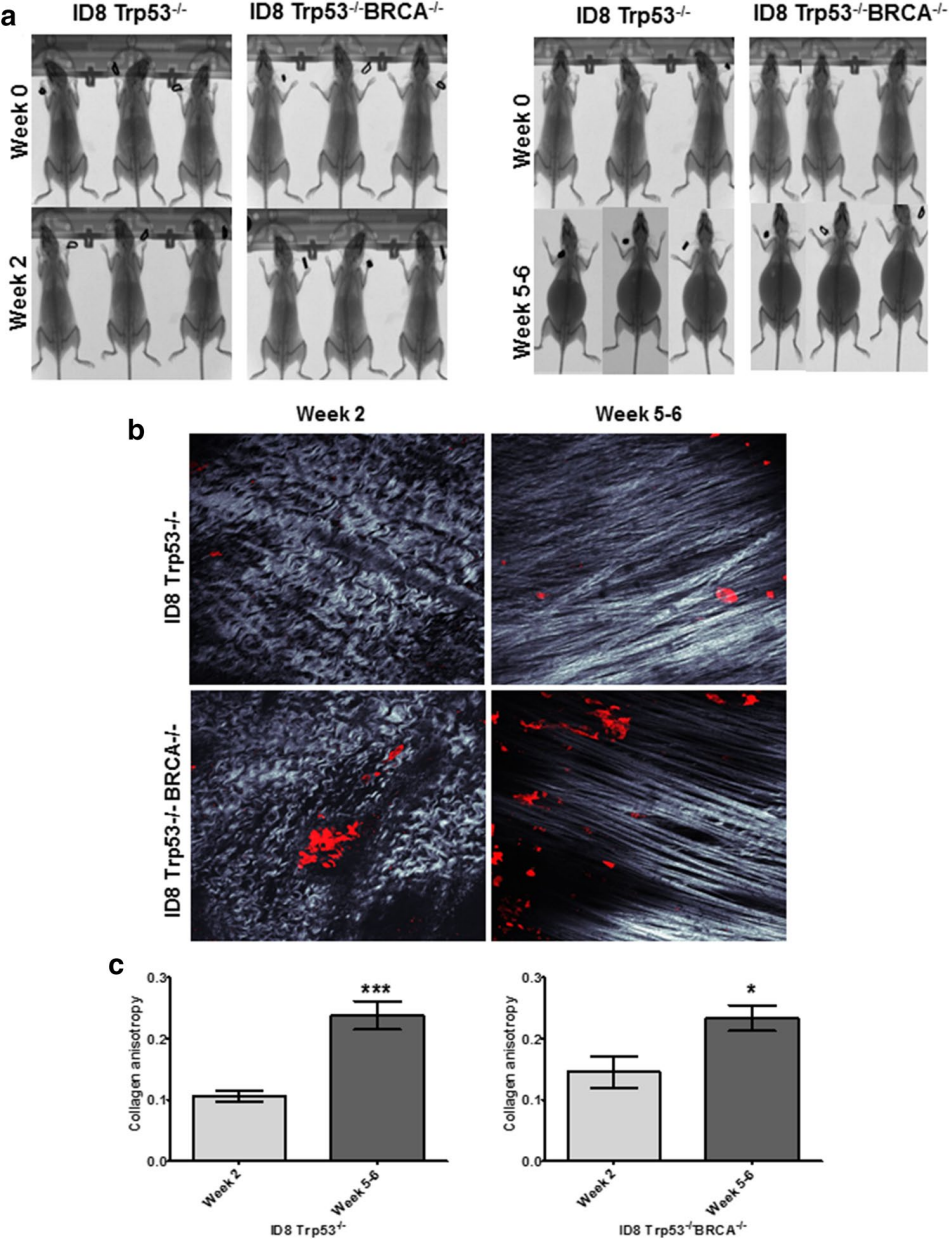
Ascites accumulation in vivo correlates with enhanced collagen anisotropy. (**A**) C57Bl/6 female mice were injected i.p. with 5 × 10^6^ RFP-tagged ID8-Trp53^−/−^ or ID8-Trp53^−/−^ BRCA^−/−^ syngeneic murine OvCa cells as indicated. MicroCT images were procured at time of injection (week 0) and time of sacrifice (week 2 or week 5–6 post-injection, as indicated). (**B**) The parietal peritoneum was dissected and prepared for combined fluorescence/SHG microscopy as described in Methods. Shown are representative examples from each cohort to show collagen fiber alignment (grey) and metastatic lesions (RFP-tagged cancer cells, red). (**C**) Collagen anisotropy was quantified using ImageJ. All results are presented as mean ± s.e.m. and *P*-values were calculated using a Student’s two-tailed *t*-test.

To determine whether ascites-induced compression alters the ultrastructure of peritoneal collagen, we injected C57Bl/6 female mice with either RFP-tagged ID8 Trp53^−/−^ or ID8 Trp53^−/−^ BRCA^−/−^, which can induce ascites formation in 5–7 weeks post injection (Fig. 7a)^33^. Mice were divided into two groups; the first group was sacrificed 2 weeks post-injection to allow i.p. metastasis without formation of ascites and the second group was sacrificed 5–6 weeks post-injection after ascites accumulation. MicroCT scans were performed to monitor ascites progression and to visualize peritoneal cavity expansion (Fig. 7a). Mice were sacrificed and the peritoneum was imaged with Second Harmonic Generation (SHG) microscopy to visualize collagen fiber alignment. Z-stacks of the peritoneal collagen were taken and pictures were evaluated to determine collagen fiber alignment (Fig. 7b). Collagen fibers in mice with i.p. metastasis in the absence of ascites were wavy with isotropic alignment (Fig. 7b, week 2). In contrast, peritoneal collagen fibers in mice bearing ascites (week 5–6) were significantly anisotropic and straight (Fig. 7b, c), indicating that ascites accumulation is associated with alterations in the sub-mesothelial matrix ultrastructure.

## Discussion

Malignant ascites accumulation in OvCa results from obstruction of peritoneal lymphatic drainage by disseminating OvCa cells, in addition to enhanced vascular permeability in the peritoneal cavity^2^. The accumulation of malignant ascites can dramatically elevate IPP from sub-atmospheric (5 mmHg) to as high as 22 mmHg^3^, changing the loading environment in the peritoneal cavity and causing compressive and shear stress. Cells adaptively transduce mechanical stimuli by converting them into biochemical signals, resulting in altered cytoskeletal organization and cellular behavior^34–36^. While the contribution of the cellular and molecular components of malignant ascites to OvCa progression is well studied^2^, the role of ascites-induced increases in IPP in OvCa progression is unknown. Herein, we aimed to model ascites-induced compressive effects on the peritoneal microenvironment and the resulting impact on OvCa peritoneal seeding.

OvCa cell metastasis to the peritoneum involves the adhesion of OvCa cells to the peritoneal mesothelial surface, migration into and anchoring within the sub-mesothelial collagen-rich matrix and proliferation to establish secondary lesions^37,38^. While tumor cells initially rapidly home to the omentum, other mesothelially-lined surfaces are populated later in metastatic progression. In models of artificial ascites, our data show rapid omental homing accompanied by significantly enhanced OvCa cell adhesion to the peritoneum. Similar results were obtained in a previous study that generated high IPP (8 mmHg) using CO_2_ pneumoperitoneum, demonstrating increased abdominal invasion of pre-injected ID8 OvCa cells in the high pressure group relative to low IPP (2 mmHg) or controls^14^. Together these data suggest that the increase in intraperitoneal adhesion observed in the current study is related to compression-induced effects rather than enhanced dispersion of cells within the peritoneal space due to enhanced fluid volumes. This is supported by experiments using ex vivo peritoneal explants which demonstrate enhanced receptivity to OvCa cell adhesion under conditions of compression.

Additional research indicated similar effects of compression on cancer cell behavior and metastatic potential^39^. For example, compression altered cytoskeletal organization and acquisition of an invasive morphology in pancreatic and breast cancer cells, leading to increased migration and invasion^40,41^. Interstitial fluid pressure regulated collective invasion in compressed breast cancer cellular aggregates^42^. Additionally, compression-induced alterations in cells in the tumor micro-environment that also contribute to disease progression have also been reported. This is exemplified by a study demonstrating that compression activated pancreatic fibroblasts, which in turn promoted pancreatic cancer cell migration^43^. Here, we show that compression induced dramatic alterations in peritoneal MC morphology, including MC retraction and acquisition of a mesenchymal phenotype. Moreover, we previously reported compression-induced changes in OvCa multicellular aggregates correlated with upregulation of epithelial-mesenchymal transition (EMT)-associated genes^11^. Together these data suggest that ascites-induced compression may play a role in mesothelial-mesenchymal transition^21,44^. A limitation of the current study is the lack of information regarding the uniformity of ascites-induced compression throughout structures of the peritoneal cavity impacted by OvCa metastases. Given the high degree of complexity of this compartment, future studies employing computational models that take into account peritoneal fluid dynamics to assess regional differences in compressive loading are warranted.

In addition to inducing ultrastructural changes that result in a more receptive peritoneal surface, novel findings show the induction of TNTs under compression in cultured cells, in intact murine peritoneum ex vivo and in vivo*,* and in human peritoneum from OvCa patients. Cells form TNTs as an intercellular communication conduit to withstand environmental stress, including metabolic stress and inflammatory conditions, via the intercellular transport of cytoplasmic organelles and molecules that promote survival^45, 46^. To the best of our knowledge, this is the first report of mechanically-regulated TNT induction in intact tissues. Mitochondrial trafficking through TNT has been shown in several cell types and can contribute to drug resistance and metabolic reprogramming in cancer cells^24–26,47^. Our results indicate that compression-induced TNTs mediate the transfer of mitochondria from MC to OvCa cells, which may contribute to OvCa cell survival under compression. TNT-mediated mitochondrial transfer can change the behavior and fate of the recipient cells^27^. A previous study determined that TNT-transferred mitochondria from mesenchymal stem cells to breast cancer cells enhanced basal and maximal oxygen consumption, reduced glycolysis and lactate production and increased the concentrations of both the endogenous mitochondrial DNA and the produced ATP^48^. Also, ATP levels increased after TNT-mediated mitochondria transfer from bone marrow stromal cells to acute myeloid cells during chemotherapy, leading to myeloid cell survival^47^. In light of these observations, future investigations will address the impact of TNT-mediated mitochondrial transfer from MC to OvCa cell on OvCa cellular metabolism and survival.

Mechanical stimuli can remodel the extracellular matrix, impacting tumor cell behavior^30,41,49–51^. Normal peritoneal tissues exhibit a random, wavy and isotropic arrangement of collagen fibers, while collagen fiber remodeling into an organized, straight and anisotropic alignment is observed under pathologic conditions and in several epithelial tumors which has been termed ‘tumor-associated collagen signatures’ (TACS)^30,52^. Anisotropic collagen fibers facilitate cancer cell motility and invasion to the surrounding stroma^53,54^. Here, we show that ascites accumulation in two different in vivo OvCa murine models is associated with an alteration in the alignment of peritoneal collagen fibers to a linear anisotropic arrangement. Together these data suggest that ascites-induced compression may contribute to OvCa progression by promoting OvCa cell adhesion to peritoneum, altering the integrity of the peritoneal MC monolayer, and enhancing interactions between OvCa cells and peritoneal MC via TNT formation. While results implicate Wnt5a as a potential molecular mediator, additional mechanistic studies are needed to elucidate the mechanosensory receptors and/or pathways that mediate the observed effects and to modulate the activity of these effectors in relevant pre-clinical models of metastatic disease.

## Materials and methods

### Cell culture and animal studies approval

The epithelial ovarian carcinoma cell lines OVCAR5, and OVCAR8 cells were obtained from American Type Culture Collection (Manassas, VA) and were maintained in Dulbecco's Modified Eagle Medium (DMEM) medium, containing 10% Fetal Bovine Serum (FBS; Gibco), 1% Penicillin/Streptomycin (Pen/Strep; Lonza) and 1% Non-Essential Amino Acids (NEAA) (Gibco). Dr. I. McNeish, Glasgow, UK, generously provided the C57Bl/6 syngeneic mouse ovarian cancer cell line (ID8) with a *TRP53* gene deletion (designated ID8-Trp53^−/−^), or both *TRP53* and *BRCA* genes deleted (designated ID8-Trp53^−/−^ BRCA^−/−^). These cells were tagged with red fluorescent protein (RFP) and maintained as previously described^37,55^. Primary human peritoneal mesothelial cells (Zen-Bio, Cary NC) and the normal human peritoneal mesothelial cell line LP9 (Coriell Aging Cell Repository) were maintained in Medium 199, Ham 12 medium, supplemented with 15% FBS, 10 ng/mL Epidermal Growth Factor (EGF), 400 ng/mL Hydrocortisone, 1% Penicillin/Streptomycin (Pen/Strep; Lonza), 1% L-GlutMAX (Thermo Fisher Scientific) and 1% HEPES. All cells were maintained at 37 °C, 5% CO_2_ in humid air. All murine studies were approved by the Institutional Animal Care and Use Committee, University of Notre Dame and were conducted in accordance with relevant guidelines and regulations of this committee.

### Human specimens

De-identified fresh peritoneum tissue was obtained through University of Kansas Cancer Center’s Biospecimen Repository Core Facility under Institutional Review Board approved protocol HSC#5929, abiding with the US Common Rule, and studies using these tissues were conducted in accordance with the relevant guidelines and regulations of this committee. Written patient consent was obtained for use of the specimen for research purposes. At the time of cytoreductive surgery, once optimal debulking was achieved, grossly normal appearing peritoneum was identified and a 2 cm diameter section removed. The peritoneum was transferred to the laboratory immediately upon retrieval from the patient, rinsed in sterile PBS, and marked with a tissue marker to indicate the outer side of the peritoneum. The tissue was then placed in SEM fixative solution (below) and incubated at 4C for 2–3 days prior to further processing as described below.

### X-Ray MicroCT scans

The microCT experiments were conducted using an Albira CT System (Bruker Xtreme In Vivo Imaging system, Billerica, MA, USA) in the Notre Dame Integrated Imaging Facility as previously described^56^. Mice were first anesthetized with isoflurane (2.5% flow rate) prior to imaging. Scans of the mice were performed with a FOV of 160 mm at low dose CT intensity (0.2 mA) and a high CT voltage (45 kVp).

### Artificial ascites in vivo adhesion assay

To model the effect of ascites on OvCa cell adhesion to peritoneum, C57Bl/6 female mice were i.p injected with 5 × 10^6^ RFP-tagged OVCAR5 or OVCAR8 in 5 mL or 10 mL PBS as an artificial ascites model or in 1 mL PBS as control. Mice were imaged using microCT, as described above, to demonstrate abdominal distension. The IPP changes correlated with these injection volumes is unknown. Mice injected with RFP-tagged OVCAR5 or OVCAR8 were euthanized by CO_2_ inhalation after 5 and 8 h, respectively, followed by cervical dislocation and then rapidly dissected using a ventral midline incision. In some experiments, only PBS (5 mL or 10 mL) was injected in the absence of OVACR cells and peritoneal tissues were processed for scanning electron microscopy as described below. After skin removal, the parietal peritoneum was dissected and vigorously washed in PBS (five times) and mounted onto a glass coverslip. Adherent cells were imaged with either the Echo Revolve fluorescent microscope or EVOS FL digital inverted fluorescence microscope and cells were counted manually using ImageJ software. The assay was performed in three experimental replicates and repeated in three biological replicates for all conditions.

### Fluorescence microscopy

To evaluate the effect of compression on cell morphology, MC were cultured on 22 mm^2^ glass coverslips coated with type I collagen (10 µg/mL) and placed into the foam sample holders of six-well BioPress culture plates with silicone elastomer well bottoms (Flexcell International Corporation, Hillsborough, NC, USA). Fresh complete culture medium (4 mL) was added to each well of a BioPress plate and stationary platens were inserted into the culture plate wells of a Flexcell Compression Plus System. Platen heights were empirically tested and adjusted according to the specific parameters of each sample condition and the compression baseplate was assembled according to the manufacturer’s specifications. Samples were incubated at 37 °C in 5% CO_2_ under static compression (~ 3 kPa; ~ 22 mmHg) applied for the indicated time points for each experiment. Control samples were incubated in BioPress plates and placed in the same incubator, with no compression applied. Coverslips were washed twice with PBS and fixed with 4% paraformaldehyde in 0.12 M sucrose in PBS for 30 min at room temperature. Non-specific binding was blocked with 5% normal goat serum in PBS for 1 h at room temperature. Coverslips were then incubated with Phalloidin488 (1:100) (Thermo Fisher Scientific) in 1% normal goat serum in PBS for 20 min in room temperature and rinsed thrice for 5 min with PBS. After washing, cells were air-dried, mounted with VECTASHIELD Mounting Media with 4′,6-diamidino-2-phenylindole (DAPI) (Vector laboratories, Burlingame, CA), and imaged with a Leica DM5500 fluorescence microscope (Leica, Biosystems, Inc.)^57,58^.

### Compression of ex vivo murine peritoneum explants

C57Bl/6 female mice were euthanized by CO_2_ inhalation followed by cervical dislocation and then rapidly dissected using a ventral midline incision. After skin removal, the parietal peritoneum lining the ventral abdominal wall was dissected to remove a 1.2 × 1.2 cm^2^ piece of peritoneal tissue immediately adjacent to the midline in the lower two abdominal quadrants. The tissue explants were placed into the foam sample holders of six-well BioPress culture plates with silicone elastomer well bottoms (Flexcell International Corporation, Hillsborough, NC, USA). Fresh complete culture medium (4 mL) was added to each well of a BioPress plate, stationary platens were inserted into the culture plate. Samples were incubated at 37 °C in 5% CO_2_ under static compression (~ 3 kPa; ~ 22 mmHg) applied for 1 h. The tissue explants were fixed and processed for scanning electron microscopy imaging as described below. The assay was performed in three experimental replicates and repeated in three biological replicates. Peritoneal tissues from three mice were used in the experiment and TNT were quantified in 10 images per mouse peritoneum using ImageJ. Data are presented as mean ± s.d. or standard error of the mean (s.e.m.). Comparison between groups was performed using Student two-tailed t-test to determine *p* values. P value < 0.05 was considered significant.

### Scanning electron microscopy

Murine peritoneal explants were dissected from 4–6 month old C57Bl/6J mice as described above and processed for scanning electron microscopy (SEM). Tissues were fixed for SEM using a fixative containing 2% glutaraldehyde and 2% paraformaldehyde in 0.1 mol/L cacodylate, pH 7.35, for one hour. Specimens were then washed with 2-mercaptoethanol (2-ME) buffer (0.1 mol/L sodium cacodylate, pH 7.35, containing 0.13 mol/L sucrose, 0.01 mol/L 2-ME) three times for 20 min each wash prior to fixation with osmium tetroxide (2% in cacodylate buffer) with microwave processing. After rinsing 3 × 5 min with cacodylate buffer and washing with ultrapure water 3 × 5 min, tissues were dehydrated in a graded series of ethanol. Following dehydration, specimens were subjected to critical point drying and placed on carbon stubs for sputter-coating with platinum. Samples were examined using a FEI-Magellan 400 Field Emission Scanning Electron Microscope in the Electron Microscopy core of the Notre Dame Integrated Imaging Facility^11,58^.

### Ex vivo adhesion assay

To evaluate the potential effect of compression on adhesion of OvCa cells to peritoneum, an ex vivo peritoneal explant assay was used^59^. Mice were first euthanized by CO_2_ inhalation followed by cervical dislocation and then rapidly dissected using a ventral midline incision. A 1.2 × 1.2 cm^2^ piece of tissue was dissected from the parietal peritoneum lining the ventral abdominal wall. The tissue explant was then placed mesothelial-side up in BioPress culture plates. RFP-OVCAR5 and RFP-OVCAR8 cells (5 × 10^4^) were added to the explant and incubated for 30 min under static compression (~ 3 kPa, ~ 22 mmHg), at 37 °C. The peritoneal explants were then washed three times in PBS, fixed and processed for scanning electron microscopy, as described above. TNT were quantified in 10 cells under control or compressed conditions using ImageJ. Experiments were conducted in triplicate with three biological replicates. Data are presented as mean ± s.d. or standard error of the mean (s.e.m.). Comparison between groups was performed using Student two-tailed t-test to determine *p* values. P value < 0.05 was considered significant.

### Mitochondria transport in LP9-OvCa cell co-culture

Mitochondria in GFP-tagged LP9 cells were labeled with MitoTracker Red CMXRos according to manufacturer protocol (ThermoFisher Scientific) then co-cultured with either OVCAR5 or OVCAR8 cells at 1:1 ratio atop of rat tail collagen I-coated coverslips (10 µg/mL). Co-cultured cells were then placed in Flexcell compression plates under compression (~ 3 kPa; ~ 22 mmHg) for 24 h. Cells were washed in PBS, fixed and imaged with a Leica DM5500 fluorescence microscope (Leica, Biosystems, Inc.).

### Fluorescence/SHG imaging of murine peritoneal collagen

Female C57Bl/6 mice were i.p injected with 5 × 10^6^ RFP-tagged ID8-Trp53^−/−^ or ID8-Trp53^−/−^ BRCA^−/−^ cells. Mice were divided into two groups of 3 mice each. One group was euthanized 2 weeks post injection to allow formation of peritoneal metastasis without ascites formation; the second group was euthanized 5–6 weeks post injection after formation of peritoneal metastasis and accumulation of ascites (6–9 mL). Mice were sacrificed by CO_2_ inhalation followed by cervical dislocation. The parietal peritoneum was dissected, rinsed with PBS and placed between coverslips for imaging with the mesothelium side facing the objective (25X XLPlanN, 1.05na WATER) of the 2-Photon confocal microscope (Olympus FV1000, software FLUOVIEW FV1000). Using a Mai Tai DeepSee titanium-sapphire 690–1040 nm laser, RFP-tagged ID8 metastatic implants and peritoneal collagen were visualized using the RFP and SHG signals, respectively. At 12% laser power, the 2-photon laser was set to 860 nm and emission was simultaneously collected at 425–465 nm and 575–625 nm for SHG and RFP, respectively. Quantification of fiber anisotropy from SHG images of collagen was done using the FibrilTool plugin in ImageJ^60^. Data are presented as mean ± standard error of the mean (s.e.m.).

### Statistical analysis

All experiments were conducted in a minimum of three independent replicates. The statistical analysis of the data was done using GraphPad Prism software or Excel software. Data are presented as mean ± s.d. or standard error of the mean (s.e.m.). Comparison between groups was performed using Student two-tailed t-test to determine *p* values. P value < 0.05 was considered significant.

## Supplementary information

Supplementary information.
