# Supplementary material for: Ascites-induced compression alters the peritoneal microenvironment and promotes metastatic success in ovarian cancer
Source: Sci Rep. 2020 Jul 17;10:11913. doi: 10.1038/s41598-020-68639-2 (PMC7367827; doi:10.1038/s41598-020-68639-2)

SUPPLEMENTAL DATA

Ascites-Induced Compression Alters the Peritoneal Microenvironment and Promotes Metastatic Success in Ovarian Cancer

Marwa Asem[1,2], Allison Young[2], Carlysa Oyama[2], Alejandro ClaureDeLaZerda[2], Yueying Liu[1,2], Matthew. J. Ravosa[2,3], Vijayalaxmi Gupta[4], Andrea Jewell[4], Dineo Khabele[4] and M. Sharon Stack[1,2*]

Supplemental Methods: Human peritoneal mesothelial cells (LP9) or human ovarian cancer (OvCa) cells (OVCAR5, OVCAR8) were cultured as described and subjected to static compression (~22 mmHg) using a Flexcell Compression Plus system as described in the main manuscript. RNA isolation, qRT-PCR, and western blotting were carried out as described in [57]. Fluorescence and scanning electron microscopy was carried out as described in the main manuscript.

Supplemental Figure 1. Compression induces WNT5A expression and Wnt5a modulates cell surface nanoscale projections. **(a)** LP9, OVCAR8 and OVCAR5 cells were subjected to static compression (~22 mmHg, 1h) followed by lysis, RNA isolation and qRT-PCR analysis of WNT5A expression levels. Experiments were performed in triplicate and results show relative expression of compressed:control. **(b)** LP9, OVCAR8 and OVCAR5 cells were subjected to static compression (~22 mmHg, 6h) followed by lysis. Lysates were electrophoresed on SDS-polyacrylamide gels, electroblotted to immobilon and probed with anti-Wnt5a and a peroxidase-conjugated secondary antibody. The full length gel with molecular weight markers is shown in panel **b**'. Note the presence of non-specific bands, the intensity of which are not altered by compression. **(c)** OVCAR8 cells were cultured in serum free medium overnight then incubated with exogenous Wnt5a (0.4 ug/ml) for 24 h prior to fixation and staining with Phalloidin488 and DAPI. Cells were imaged with a Leica DM5500 fluorescence microscope. Scalebar 100 um. **(d)** OVCAR5 cells were cultured in serum free medium overnight then incubated with exogenous Wnt5a (0.4 ug/ml) for 24 h prior to fixation and processing for scanning electron microscopy using an FEI-Magellan 400 Field Emission Scanning Electron Microscope. Scalebars 4-10 um as indicated.

Asem et al., Suppl. Fig. 1

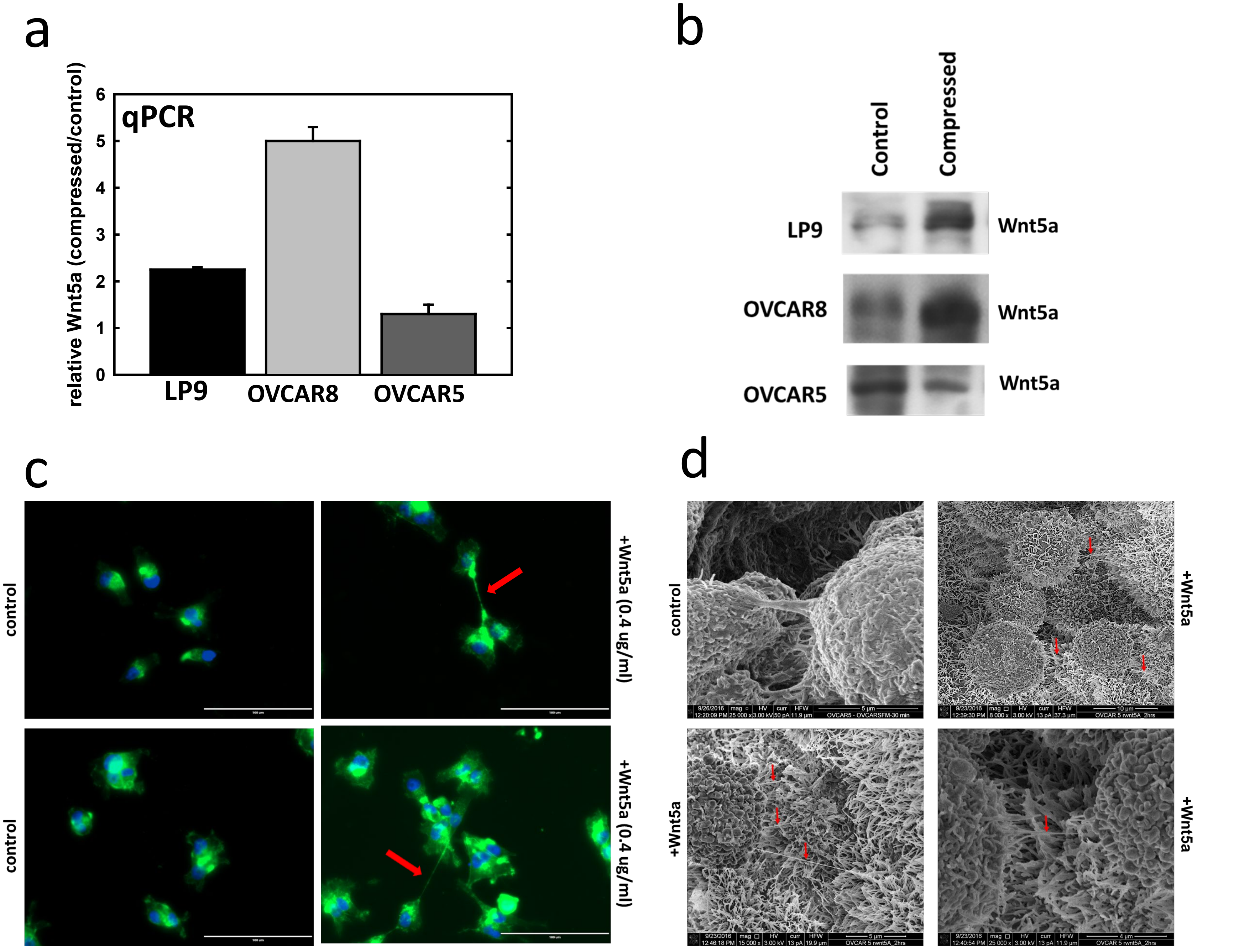

Asem et al., Suppl. Fig. 1b’

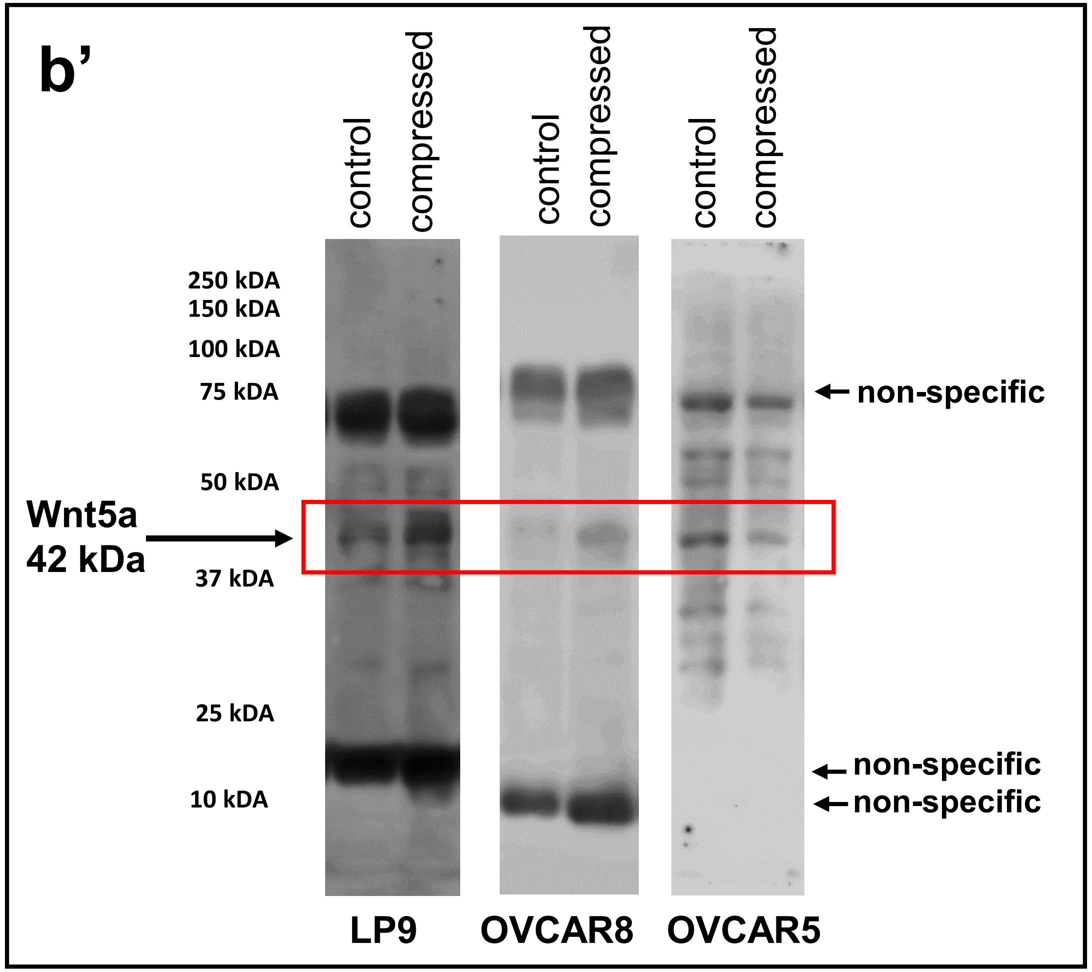

Supplement: Supplementary file 1 — Supplementary information. [file 41598_2020_68639_MOESM1_ESM.pdf]
